# Hygiene and the Elementary School Teacher

**Published:** 1920-06-12

**Authors:** 


### HYGIENE AND THE ELEMENTARY SCHOOL TEACHER.

By a SOCIAL WORKER.

During a collective conference of forty educational associations at University College, two societies combined to arrange a course of twelve lectures with a special view to the new regulations for hygiene teaching in training colleges.

Now this syllabus is very important. It was a serious defect in the earlier teaching, one which may be found reflected in a mass of text-books published during a number of years, that hygiene teaching in elementary schools consisted largely of elementary anatomy and physiology, with infinite detail and pictorial illustration of processes such as digestion and circulation. Mothers were not sympathetic. "It don't do 'em no good, and besides, it's rude," they said. Matters were not much mended by increasing study of proteids and vitamines as war diet became a pressing question. Now, however, there seems hope, and this syllabus encourages it, that our authorities will really seek the best means of ranging the children on the side of the angels in a war against dirt, disease, and depravity. It is the tragedy of our time that we have to increase and multiply such things as infant-welfare centres and mother schools, besides the host of other remedial measures, till such time as a rational education makes such things unnecessary. Certainly without it neither improved housing nor a good deal of other effort for betterment will be of use. You cannot finally abolish the slum till you have abolished the slum-maker.

### The Principles of Health.

The Board of Education expressly state that the syllabus for training the young teacher is not intended as a syllabus for school use later. It is meant to give a wide survey of the subject and of its relations with other subjects, thus fitting the student to teach the principles of a healthy life, both directly and indirectly. This idea that more might be done in the way of indirect teaching came up several times in the lectures and following discussions, as in Dr. Constance Long's remark that sex hygiene might be taught in the history lesson if it were explained that a king's love affair influenced the Reformation in England, and in the Scripture lesson if David's error were not slurred over but used with purpose. The Board, however, have made two distinct pronouncements on two pressing questions. Sex hygiene remains an optional subject for the College course, though they "suggest" that it should be included. But they "are satisfied that detailed teaching of sex hygiene is inappropriate in the public elementary school." Evidently, however, the mass of teachers are not so satisfied. In consequence of the questions asked and desire shown, the societies responsible for the lectures (the clumsily-named National Association for the Prevention of Infant Mortality and the Civic Education League) arranged at once for a series of lectures by Miss Norah March on methods of instruction. Miss March's lecture with lantern slides, showing how she would approach the subject through Nature study, interested a large audience. The extreme view was represented by the head-master of a mixed country school, who asked whether such instruction could be given to his children collectively, and discussed freely throughout the school.

The second pronouncement of the Board of Education in the preface to its syllabus is that "equally inappropriate" is direct teaching on venereal disease in the school. This is easily said, and no doubt we should all heartily agree but for the undoubted fact that boys and girls are learning from each other, from the newspapers, and from the talk of their elders a great deal about this unpleasant subject, and precisely those things about it which they had better not hear. Is it, then, really wise to prevent the people who could tell them the right things in the right way from doing so? The final lecture of the course at University College was given by Mr. E. R. T. Clarkson under the title, "Some Modern Problems of Sex Ethics," and the audience had reason to be grateful for the wholesome tone he gave to the subject.

### A Refreshing Tone.

It is refreshing to be told that boys—and girls—may be taught two things especially: First, the gross selfishness of satisfying a personal instinct at the cost of another. For it cannot fairly be said that women are duly repaid by gifts of money or luxury. Secondly, that the lives, well known to doctors, of thousands of men who refrain from adding to the social evil, even if obliged to marry late, are a reality and an example. Unfortunately, boys hear little of them, as they hear little of the thousands of bank clerks who do not gamble and abscond with employers' money. Lecky's piteous description of the "most mournful and even terrible figure," the "guardian of the purity of others," might well be pointed out to those with whom in the last resort it must rest to create or to abolish her. Miss Alice Woods and Dr. Constance Long spoke on the teacher's part and opportunities in dealing with girls who come under their care during that transition period in which they may develop abnormal symptoms. Here, again, fresh air was let in to matters that have been kept unwholesomely covered up. Fear, as Mr. Clarkson said, is of no use to anybody. It is the simple, straight, healthy view of life as the great adventure that is required for youth in days of freedom and new opportunity. Fear, curiosity which begins by being natural but becomes dangerous in secrecy, selfishness, and that love of luxury which leads to deferred marriage and the refusal of family responsibility—these are several germs of social harm to be fought and killed. Students trained under the Board's syllabus should learn this, and will then be better able to help the children.

